# The relationship between muscle mass changes and protein or energy intake in critically ill children: A systematic review and meta‐analysis

**DOI:** 10.1002/jpen.2715

**Published:** 2024-12-24

**Authors:** Lewis J. Stacey, Frederic V. Valla, Chao Huang, Paul Comfort, Corinne Jotterand Chaparro, Lynne Latten, Lyvonne N. Tume

**Affiliations:** ^1^ Edge Hill University Ormskirk UK; ^2^ Pediatric Intensive Care, Hospices Civils de Lyon Lyon France; ^3^ Institute for Clinical and Applied Health Research and Hull York Medical School University of Hull Hull UK; ^4^ Directorate of Psychology and Sport University of Salford Salford Greater Manchester UK; ^5^ Department of Nutrition and Dietetics, Geneva School of Health Sciences HES‐SO University of Applied Sciences and Arts Western Switzerland Geneva Switzerland; ^6^ Paediatric Critical Care Dietician, Alder Hey Children's NHS Foundation Trust Liverpool UK; ^7^ Pediatric Intensive Care Unit, Alder Hey Children's NHS Foundation Trust Liverpool UK

**Keywords:** child, intensive care, muscle, pediatric, sarcopenia

## Abstract

Survivorship after pediatric critical illness is high in developed countries, but many suffer physical morbidities afterwards. The increasing focus on follow‐up after critical illness has led to more pediatric studies reporting muscle mass changes (using ultrasound), albeit with different results. A systematic literature review was undertaken examining muscle mass changes, assessed by ultrasound of the quadriceps femoris muscle in children who are critically ill. Secondary objectives were to determine if muscle mass was associated with protein intake and/or energy. Databases were searched in July 2024. Eligible experimental or observational studies, published from January 2010 to July 2024 and including children who are critically ill that were aged between ≥37 weeks' gestational age and 18 years who were admitted to the pediatric critical care unit were included. The Joanna Briggs Institute for observational studies critical appraisal instrument was used to assess studies for methodological quality. One hundred and thirty‐five studies were screened, and eight prospective cohort studies were included, involving 411 children. Overall, muscle mass changes reported in seven out of eight of the papers showed a pooled mean muscle mass loss of 8.9% (95% confidence interval [CI] 6.6–11.4) from baseline to days 5–7. Five of the eight publications defined muscular atrophy as a decrease in muscle mass of >10%. Using this cutoff, 92 (49.2%) children developed muscular atrophy during their PICU admission. Overall, muscle mass decreased by nearly 10% during a child's first week in PICU, with almost half of children developing muscular atrophy during their admission.

## INTRODUCTION

Mortality in pediatric intensive care (PICU) is very low in developed countries.^1^ Most children now survive critical illness but often suffer from ongoing physical, cognitive, and psychosocial morbidities, now known as PICS‐p,^2^ which can afflict up to 50% of children.^3^ After discharge from PICU, studies have reported that children have difficulties in their physical function ranging from mild to severe disabilities.^4^, ^5^

Point of care ultrasound (POCUS) is a tool increasingly used in critical care for a variety of applications, including assessing soft tissue.^6^ Valla et al validated a technique of quadriceps ultrasound measurement to assess muscle mass in children, showing POCUS could reliably detect the extent of muscle mass loss in children.^7^ It is important to identify children at risk of muscle mass loss to be able to intervene earlier, with the aim of minimizing the long‐term consequences. Minimizing muscle atrophy is important as muscle wasting (using POCUS) has been increasingly reported in this population,^7^, ^8^, ^9^, ^10^, ^11^, ^12^, ^13^, ^14^ albeit with differing results. Therefore, we aimed to systematically review literature examining muscle mass changes, assessed by ultrasound of the quadriceps femoris muscle, in critically ill children. Our secondary objectives were to determine if muscle mass is associated with protein intake and/or energy intake, or other factors such as severity of illness, length of hospital stay, length of mechanical ventilation, and use of medications (including steroids and neuromuscular blockade).

## MATERIALS AND METHODS

### Protocol and registration

We undertook a systematic review following the Joanna Briggs Institute framework.^15^ The review protocol was registered in Open Science Framework^16^ and reported according to PRISMA 28 and PRISMA S29 reporting guidelines.^17^, ^18^

### Definitions

For this study we defined muscular atrophy as a >10% decrease in muscle mass measurement during the child's PICU stay.

### Outcomes and eligibility criteria

The primary outcome was muscle mass changes in the quadriceps femoris muscle in children who are critically ill. The secondary outcomes were protein intake, energy intake, and other risk factors, such as severity of illness, length of hospital stay, length of mechanical ventilation, and use of medications (such as steroids and neuromuscular blockade).

### Search strategy

A comprehensive search strategy was designed (in conjunction with an information specialist) and adapted to answer the question: what are the muscle mass changes in the quadriceps femoris muscle in children who are critically ill?

A secondary question was: what is the association of this muscle mass loss with protein and/or energy intake, or other risk factors, such as severity of illness, length of hospital stay, length of mechanical ventilation, and use of medications (such as steroids and neuromuscular blockade).

The following databases: MEDLINE, CINAHL Complete, Embase, Cochrane Library Central Register of Controlled Trials and trial registers, such as Clinicaltrials.gov and the World Health Organization's International Clinical Trials Registry. The full search strategy is shown in Table S1. The search included information sources/databases published from January 1, 2010, to July 31, 2024. This date range was chosen as the first international guidelines for POCUS in critical care were published in December 2020,^19^ so we selected a decade before this date and could find no papers published before this time.

### Study selection

This review included experimental or observational studies that included term neonates (born at ≥37 weeks' gestation) up to 18 years old who were admitted to PICU for a minimum of 48 h and had quadriceps muscle mass assessed by ultrasound. Any studies involving preterm neonates and/or adults were excluded.

All identified citations were collated and uploaded onto Rayyan software TM (Qatar Computing Research Institute) for screening, and any duplicated sources were removed. The titles and abstracts were initially screened by two independent reviewers (L. S., L. T.) for assessment against the inclusion criteria. Any potentially relevant studies were retrieved in full text for checking. Full‐text papers were then assessed against the inclusion criteria by two independent reviewers (L. S, L. T.), and any disagreements at any stage were resolved by a third reviewer (F. V.).

### Data extraction

Data was extracted into a modified version of the Joanna Briggs Institute (JBI) tool. When key data was missing, authors were contacted. Included studies were critically appraised using the JBI critical appraisal tool for analytical studies.^20^ Each study was evaluated across eight domains to assess the risk of bias. A figure illustrating the reviewers’ judgments was generated using the robvis tool.^21^

### Data analysis

The characteristics of the included studies were summarized in a summary table. The data was extracted into a Microsoft Excel file for a meta‐analysis. We utilized the metafor package in R language (version 4.2) to assess the percent loss in muscle mass during days 5–7 from baseline.^22^ This analysis was conducted by random effect model, with the level of study heterogeneity represented by *I* ^2^. Because the correlation coefficient between baseline and days 5–7 was not reported, we assumed it was zero. One study reported the study outcomes for two subgroups, which were aggregated for meta‐analysis. Three studies reported median and interquartile range (IQR) of the study outcome, we transferred them to a mean and SD, following Cochrane guidance.^23^

For outcomes where a meta‐analysis was not possible, studies were presented narratively. The data were presented by calculating an overall median muscle mass change for all the studies and presented in relation to protein intake and/or energy intake and other risk factors, such as length of stay, use of medication (such as steroids and neuromuscular blockers), and severity of illness scores. Muscular atrophy, defined as a decrease in muscle mass percentage >10%, was also summarized and presented.

## RESULTS

### Study selection

**Figure 1:**
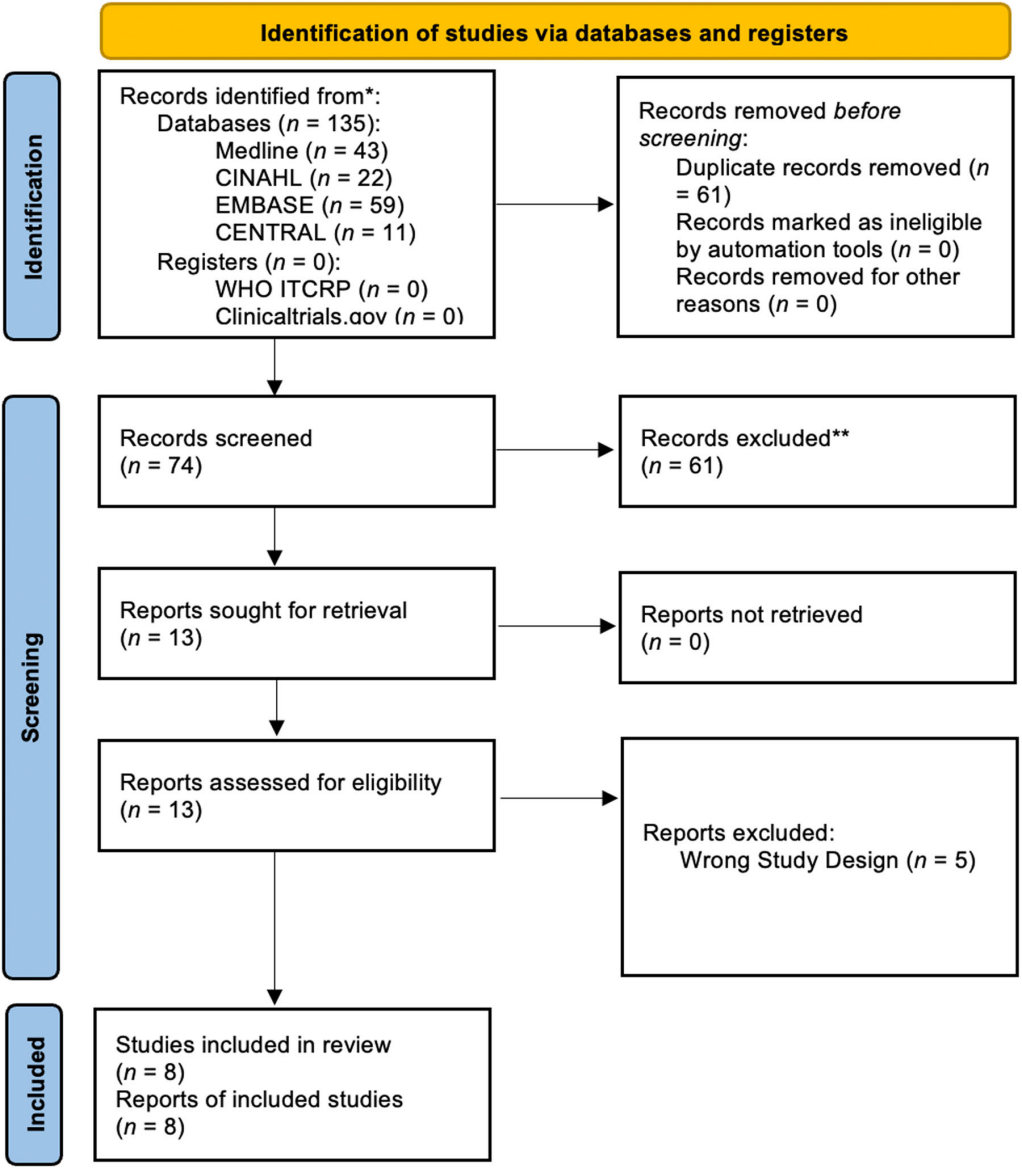
PRISMA 2020 flow diagram for the search following the search criteria.

One hundred and thirty‐five publications were identified; 61 of these were duplicates and removed. Finally, eight studies were included in this review^7^, ^8^, ^9^, ^10^, ^11^, ^12^, ^13^, ^14^ (Table 1), as illustrated in the PRISMA diagram (Figure 1). Included studies were published between 2017 and 2024, and all were prospective observational cohort studies.

**Table 1:** Summary of evidence table.

Study reference	Study aesign	Country and study duration (months)	Total number of participants recruited and number followed up, if different (%)	Study demographics: Age; sex distribution; weight; illness mortality score; adjusted z score(s)	Outcomes measured	Ultrasound method used	Data collection time frame	Muscle value results: QF thickness, QF CSA, muscle atrophy, and muscle(s) echogenicity
Valla et al^7^	Prospective cohort study; Single center	UK; Duration: November 2015 to April 2016 (5 months)	17	Age, median (IQR): 47 (5–126) months; Sex, number (%): 2 (11.8%) women, 15 (88.2%) men; Weight: 20 (7.8–29.5) kg; BMI *z* score: 0 (–0.75 to 0.7)	QF thickness, muscle atrophy, age, weight, length of PICU stay, BMI *z* score, maximum CRP score, use of sedative drugs, use of neuroblocking agents, MV duration, energy intake, protein intake	US Method: Performed with the ultrasound in B‐mode and perpendicular to the skin. The patient was placed supine and only one leg was measured, which was fully extended and positioned in neutral position. To calculate QF thickness the anterior heads of RF and VI muscles thickness were measured. Measurements were taken with patients that were either cooperative or sedated. Four measurements were gathered, two transverse measurements, and two longitudinal and averages of the two measurements were obtained; Location: Used measuring tape to identify the widest portion of the thigh, then measured the distance of this location to the superior tip of the patella and recorded this value. This area was marked with an indelible marker pen, and this was the site of all measurements; US Device: Vivid S6 or SonoSite EDGE with transducer frequencies of 9–13 MHz; Operator(s): seven operators; Reliability: Intraoperator and interoperator repeatability and reproducibility studies were conducted with seven operators to develop the technique.	Within 24 h of admission, day 5, Final measurement	Change in QF thickness from admission, %, median (IQR); day 5: −9.8 (−13.7 to 0.5); Last measurement: −13.3; (−25.4 to −8.7); QF thickness, cm, median (IQR); admission: 2.25 (1.72–2.79); day 5: 2.11 (1.36–2.43); last measurement: 1.75 (1.36–2.33); muscle atrophy (participants > 10% decrease in QF thickness); No (%): day 5: 7 (41%); last measurement: 10 (59%)
Johnson et al^10^	Prospective cohort study; Single center (PICU)	United States; Duration: June 2015 to May 2016 (11 months)	34	Age, mean (95% CI): 5.42 (3.44–7.40) years; Sex, number (%): 15 (45.5%) women; 18 (54.5%) men; BMI, mean (95% CI): 17.23 (15.76–18.70) kg/m^2^; PRISM III, mean (95% CI): 14.72 (4.00–30.00)	QF thickness, EIM‐derived fat changes, Length of PICU stay, Length of hospital stay, MV‐free days, Glucocorticoids use, Neuromuscular blockade use	US method: Measured muscle thickness with a bedside ultrasound and electrical impedance using an EIM device. Measurements were taken three times, and an average gathered; Location: Temporarily marked and regularly reinforced a position ½ distance from ASIS to patella's superior edge; US Device: Sonosite Edge II (FUJIFILM Sonosite Inc) using a 13–6 MHz 6 cm linear probe (L25); Operator(s); Reliability: Performed interrater to ensure results were reliable	Timings: Enrolment then every 5–8 days until PICU discharge	Change in quadriceps thickness, %, mean (95% CI): Day 5/6 8.62; (−15.7 to −1.54) *P* = 0.0187; Muscle atrophy (participants ≥ 10% decrease in QF thickness), No (%): 16 (53%)
de Figueiredo et al^11^	Prospective cohort study; Single center (PICU)	Brazil; Duration: July 2017 to April 2018 (9 months)	119; Subgroup 1: 55 (values measured baseline and day 7); Subgroup 2: 32 (values measured baseline, day 7 day 14)	Age, median (IQR): 12 (4.0–42.5) months; Sex, number (%): 47 (39.5%) women; 72 (60.5%) men; WAZ Score, median (IQR); −1.29 (−2.85 to 0.04), measured in <10 years (*n* = 108); BMI *Z*‐score, median (IQR); −0.62 (−2.17 to 0.24), measured in all ages; PIM2, median (IQR): 2.6 (1.3–5.6)	QF thickness, QF % change, Daily intake, MV need, Length of PICU stay, In‐hospital mortality	US Method: Used in B‐mode, with the transducer placed perpendicular to the skin. Participants' muscles were relaxed and placed in a supine position with the knee in passive extension and neutral rotation. Maximum compression was applied in the transverse section; Location: Right leg along the long axis of the anterior thigh, two‐thirds distance from the ASIS to superior patella border; US Device: Vivid Q (GE Healthcare) equipped with a linear probe 5–13 MHz; Operator(s): Single operator by a qualified pediatric ultrasound instructor; Reliability: Performed intrarater variability analysis.	Timings: Baseline, day 7, day 14	Change in quadriceps thickness, %, mean ± SD; Day 7 Subgroup 1: −12.83 ± 14.07, P < 0.001; Day 7 Subgroup 2: −13.81 ± 13.05, P < 0.001; Day 14 Subgroup 2: −16.35 ± 13.04, *P* < 0.001; QF thickness, cm, mean ± SD; Baseline Subgroup 1: 0.65 ± 0.23; Baseline Subgroup 2: 0.63 ± 0.22; Day 7 Subgroup 1: 0.56 ± 0.19; Day 7 Subgroup 2: 0.53 ± 0.17; Day 14 Subgroup 2: 0.52 ± 0.17; Muscle atrophy (participants > 10% decrease in QF thickness), No (%): 32 (58.2%) from baseline to day 7
Ong et al (2023)	Prospective cohort study; Single center (PICU)	Singapore; Duration: January 2015 to October 2018 (45 months)	73; Follow‐up: 44/73 (60.2%)	Age, median (IQR): 3.1 (0.8–9.2) years; Sex, number (%): 34 (46.6%) women; 39 (53.4%) men; Weight, median (IQR): 14.6 (7.5–30.3) kg; BMI *Z*‐score, median (IQR); −0.73 (−1.79 to 0.42); PIM3, median (IQR): 0.80 (0.40–2.20)	RF CSA, RF echogenicity, Quadriceps fat thickness, Protein intake, Energy intake, Length of MV	US Method: Measurements were taken with the child in a supine position with legs extended and neutral. Transverse B‐mode was conducted on the right leg. Images were obtained in triplicate and average values were used; Location: Halfdistance from the ASIS to the superior border of the patella, in children ≥6 years, a distance of two‐thirds from ASIS was used; US Device: LOGIQe, GE Healthcare, Chicago, IL, USA with a 5–13 MHz linear array transducer; Operator(s): Single operator; Reliability: Interrater correlation was calculated.	Timings: Within 48 h of PICU admission, then days 3, 7, and 10, PICU discharge and hospital discharge; Follow‐up: At 6–12 months after hospital discharge, median (IQR): 6.7 months (6.0–7.7)	RF CSA, % change from baseline, mean (95% CI); Day 3: −8.4 (−20.2 to 3.5) *P* = 0.147; Day 7: −6.2 (−21.8 to 9.4) P = 0.400; Day 10: −2.2 (−21.9 to 17.7) P = 0.812; RF echogenicity, % change from baseline, mean (95% CI); PICU discharge: −3.0 (−11.8 to 5.8) *P* = 0.497; Hospital discharge: −1.0 (−0.61 to 7.60) *P* = 0.346; Follow‐up: 14.7 (‐0.8 to 30.2) P = 0.063; Muscle atrophy (participants > 10% decrease in QF thickness), No (%): 41 (56.2%)
Hoffmann et al^14^	Prospective cohort study; Single tertiary care children's hospital	United States; Duration: December 2017 to December 2019 (23 months)	36	Age, median (IQR): 9.2 (2.2–16.5) years; Sex, number (%): 11 (31%) women 25 (69%) men; Weight, median (IQR): 21.4 (11.8–41.3) kg	QF thickness, Protein intake, Energy intake, Fluid output, Length of hospital stay, Use of steroids, Use of paralytic agents, Length of MV	US Method: Measured the combined thickness of RF and VI. Ultrasound images were gathered in B‐mode with copious gel, and the patient was placed in the supine position their leg was extended and the transducer was placed perpendicular to the skin. This was repeated for both short axis and long axis to obtain two of each image (4 in total), which was repeated every 3 days until discharge or study day 15; Location: Two of five of the distance from the ASIS to the superior patella border, a surgical pen marked the site; US Device: General Electric Logiq S8 with 9L‐D 3.1–10 MHz linear probe or General Electric Nextgen Logiq e with L4‐12t‐RS 4.2–13 MHz linear probe; Operator(s): Five operators (two pediatric radiologists, two pediatric critical care attendings, and one pediatric resident); Reliability: Performed intraclass correlation which showed no statistical difference.	Timings: Within 72 h of ICU admission, then repeat every 72 h	Change in quadriceps thickness, %, mean ± SD; Day 3: −4.5 ± 6.6; Day 6: −8.2 ± 8.0
Jain et al^9^	Prospective cohort study; Single center (PICU) of a tertiary care hospital	India; Duration: January 2018 to June 2019 (17 months)	73	Age, median (IQR): 72 (24–108) months; Sex, number (%): 22 (37.9%) women; 36 (62.1%) men; Weight, median (IQR): 15 (10–23) kg; WAZ score, median (IQR); −1.72 (−2.71 to 0.11); PIM2 Score, mean (SD): 16.17% (18.66)	QF thickness, QF echogenicity, RF echogenicity, VI echogenicity, Calorie intake, Length of PICU stay, Duration of MV	US method: Took measurements with the patient supine with leg extended in neutral rotation and performed using B‐mode and the transducer was placed perpendicular to the skin; Location: Right thigh at a point midway between the ASIS and superior border of the patella, which was marked with an indelible pen; US Device: CX‐50, Philips Co, using a linear transducer (3–12 MHz frequency); Operator(s): Single investigator, performed interobserver study in 20 patients	Timings: Days 1, 3, and 7 of ICU stay	Change in QF thickness from day 1, cm, median (IQR); Day 3 0.09 (−0.1 to 0.46) *P* = 0.039; Day 7 −0.09 (−0.32 to 0.51) P = 0.35; QF thickness, cm, median (IQR); Day 1 1.58 (1.29–2.04); Day 3 1.82 (1.4–2.14); Day 7 1.66 (1.43–2.08); VI echogenicity, median (IQR); Day 1 35.52 (24.44–42.77); Day 3 36.21 (27.08–46.16); Day 7 40.92 (26.99–47.88); RF echogenicity, median (IQR); Day 1 25.88 (16.16–40.53); Day 3 26.64 (20.73–37.6); Day 7 30.48 (24.80–45.67)
Valverde Montoro et al^13^	Prospective cohort study; Single center (PICU)	Spain; Duration: January 2020 to June 2021 (17 months)	41	Age, median (IQR): 6 (0–22) months; Sex, number (%): 22 (53.7%) women, 19 (46.3%) men; Weight, median (IQR): 5.7 (4–12) kg; BMI *z* score, median (IQR): −0.92 (−4.13 to 2.16); PRISM III, median (IQR): 7.5 (4–12)	QF thickness, Protein intake, Energy intake, Length of PICU stay, Length of hospital stay, Use of neuroblocking agents, Use of systemic corticosteroids, Length of MV	US Method: Followed the Valla et al method to measure the combined thickness of RF and VI. The patient was in the supine position and their right leg was extended and in neutral position. Performed in B‐mode with copious gel. An average of three measurements was taken; Location: Right thigh at a point midway between ASIS and superior border of the patella, which was marked with an indelible marker pen; US Device: SonoSite M‐turbo portable ultrasound with a 12 MHz linear transducer; Operator(s): Two operators who are specialists in pediatric critical care; Reliability: Both intraoperator and interoperator studies were conducted.	Timings: Within 24 h after starting MV, Repeated at 72 h, 1 week then weekly until extubated	Change in quadriceps thickness, %, median (IQR); Day 3: −4.67 (−13.4 to −0.59) *P* < 0.001; Day 7: −13 (−24 to −0.5) P < 0.001; QF thickness, cm, median (IQR); Day 1: 1.56 (1.38–2.05); Day 3: 1.45 (1.3–1.95); Day 7: 1.37 (1.13–1.71); Muscle atrophy (participants > 10% decrease in QF thickness), No (%): 23 (56%)
Tume et al^8^	Prospective cohort study; Single center (PICU)	UK; Duration: September 2021 to December 2022 (15 months)	34; Follow‐up: 11/34 (36%)	Age, median (IQR): 6.65 (0.47–57.5) months; Sex, Number (%): 11 (32.4%) women; 23 (67.6%) men; Weight, median (IQR): 4.42 (3.4–22.8) kg; WAZ score, mean ± SD and median (IQR); −0.92 (0.0335) and −0.74 (−1.59 to 0.087); PIM3 Score, median (IQR): 0.010 (0.005–0.038)	QF thickness, QF CSA, Protein intake, Energy intake, Exposure to steroids, Exposure to neuromuscular blockade, Inflammatory markers (CRP), Length of PICU stay, Length of hospital stay, Duration of MV, Mortality	US method: Used Valla et al method; Location: Quadriceps; US Device: Sonosite (Fujifilm); Operator(s): 99% of measurements were taken by one trained operator; Repeatability: Mean of four measurements (in two different incidences)	Timings: Day 1, 3, 5, 7, and 10 of PICU stay (day 10 was excluded because of limited sample size), PICU discharge, Hospital discharge; Follow‐up; At 3 months	QF thickness, %, mean (95% CI); Day 1 1.86 (0.93–2.78); Day 3 2.03 (1.16–2.91); Day 5 2 (1.06–2.93); Day 7 1.84 (1.05–2.63); PICU discharge 1.65 (1.01–2.28); Hospital discharge 1.91 (0.98–2.84); Follow‐up 2.22 (1.35–3.09); QF CSA, mean (95% CI) %; Day 1 2.04 (−0.37 to 4.44); Day 3 2.46 (−0.09 to 5.02); Day 5 2.44 (−0.4 to 5.28); Day 7 1.76 (−0.19 to 3.72); PICU discharge 1.62 (−0.17 to 3.41); Hospital discharge 2.4 (−0.01 to 4.8); Follow‐up 3.02 (0.08–5.95); Muscle atrophy (participants > 10% decrease in QF thickness), No (%): 15 (44%)

Abbreviations: ASIS, anterior superior iliac spine; BMI, body mass index; CI, confidence interval; CRP, C‐reactive protein; CSA, cross‐sectional area; EIM, electrical impedance myography; IQR, interquartile range; MV, mechanical ventilation; PICU, pediatric intensive care unit; PIM2, Pediatric Index of Mortality 2; PIM3, Pediatric Index of Mortality 3; PRISM, pediatric risk of mortality; QF, quadriceps femoris muscle; RF, rectus femoris muscle; SD, standard deviation; UK, United Kingdom; US, ultrasound; VI, vastus intermedius muscle; WAZ, weight adjusted *z score*.

### Study characteristics

Overall, there were 411 children included in these studies, 164 (39.9%) female and 247 (60.1%) male. The median age was reported in seven of the eight studies and was 37.2 months (range, 6–110.4 months), and median weight, reported in six studies was 14.8 kg (range, 4.42–21.4 kg). Studies took place in the following countries: United Kingdom (2), United States (2), Brazil (1), Singapore (1), India (1), and Spain (1).

### Pooled analyses

#### Primary outcome

**Figure 2:**
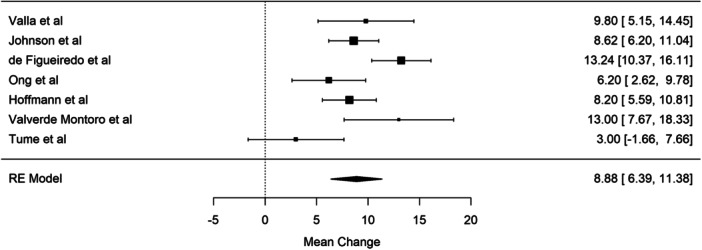
Forest plot showing the percentage mean muscle mass change for seven studies from baseline to days 5–7 after pediatric intensive care unit (PICU) admission.

**Figure 3:**
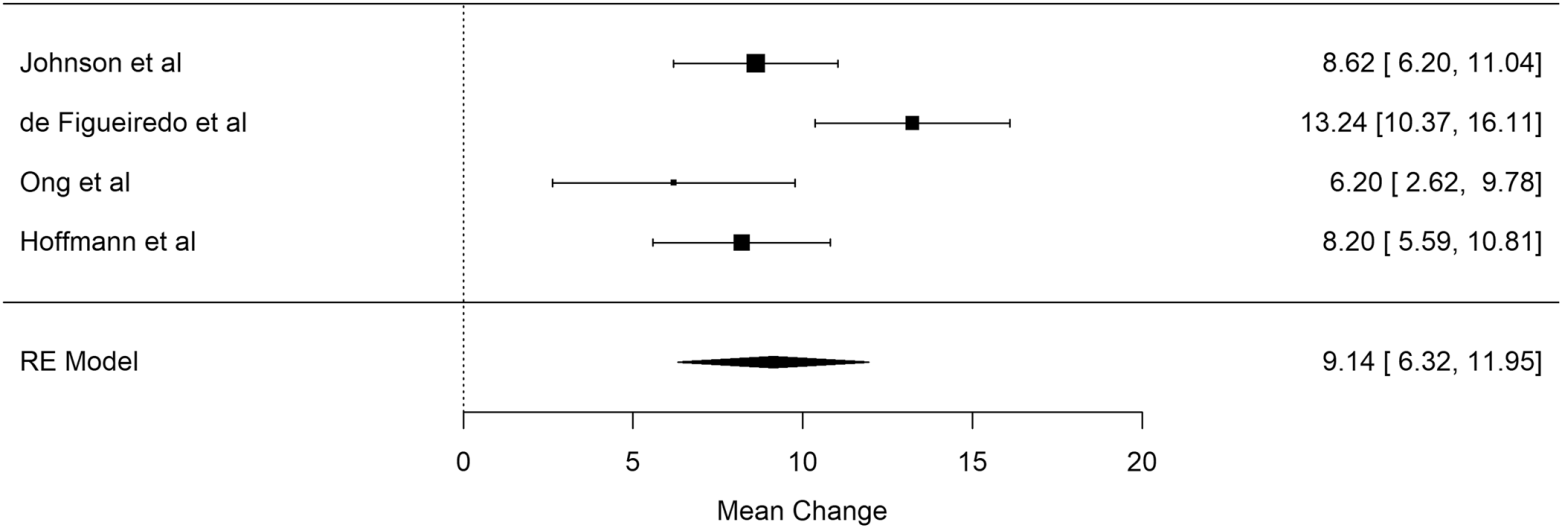
Forest plot showing the percentage mean muscle mass change for four studies from baseline to days 5–7 after pediatric intensive care unit (PICU) admission.

As shown in Table 1, the median percentage changes in muscle mass from baseline to PICU days 5–7 was measured in four of the eight studies. When we calculated the median percentage change of muscle mass for these studies, it was −9.8% (range, 3%–13%).^7^, ^8^, ^13^ In addition, the percentage change was measured in seven of the eight studies (using mean or median).^7^, ^8^, ^10^, ^11^, ^12^, ^13^, ^14^ When we pooled the results, the meta‐analysis showed a mean muscle mass loss of 8.9% (95% confidence interval [CI]: 6.4–11.4%). This was also reported as a forest plot (Figure 2). The *I* ^2^ for this random effect model was 72.3%, confirming the high level of heterogeneity. Excluding the three studies that reported median and IQR, the pooled results show a 9.1% (95% CI, 6.1–12%) muscle loss and 74.5% heterogeneity. Figure 3 reports the forest plot.^10^, ^11^, ^12^, ^14^ Five studies reported muscular atrophy, consistently defined as a decrease in muscle mass >10% of baseline. Using this definition, around half of children (*n* = 92; 49.2%) developed muscular atrophy during their PICU stay.

#### Secondary outcomes

##### Impact of protein intake

As shown in Table S2, four studies provided protein intake from days 1–7 of PICU stay.^7^, ^8^, ^11^, ^13^ All studies showed lower protein intakes compared with the minimum recommended intake of 1.5 g/kg/day for this population^24^ and a decrease in muscle mass between baseline and days 5–7 after PICU admission. However, all studies provided different variables around protein intake, which complicates the comparison. Two studies provided a percentage intake of 1.5 g/kg/day.^8^, ^11^ In the first study,^8^ the median protein intake reached 55% (IQR, 20.2–68.2%) and had a muscle mass loss of 3% (95% CI, –18.7%) on day 7. In the second study,^11^ the mean protein intake reached 72% (95% CI, 67–83%), and the mean muscle mass loss was −13.33% ±13.56%. In the study of Valla et al,^7^ the median percentage cumulative protein intake deficit at day 5 was −58.9% (IQR, –74.4 to –28.0%), and the median muscle mass change was –9.8% (IQR, –13 to –3%). The last study^13^ provided the cumulative protein intake deficit at day 5 in g/kg/day, at day 7, the authors found a mean deficit of –0.34 g/kg/day (95% CI, –1 to 0 g/kg/day) and a median change in muscle mass of –13% (IQR, –24 to –0.5%).^13^

### Impact of energy intake

Similarly, there was no consistency among studies in the definition of energy intake. Only three studies reported energy intake from days 1 to 7 of PICU admission ^7^, ^8^, ^11^ at baseline, and at days 5–7 after PICU admission, as shown in Table S3. One study defined energy intake as the percentage deficit compared with the Schofield equation, finding a median deficit of –55.3% (IQR, –64 to –16.5%) and a median change in muscle mass of –9.8% (IQR, –13.7 to –0.5%) by day 5.^7^ Tume et al.^8^ defined energy intake as a percentage of goal intake based on the Schofield equation, in this study they found a median energy intake of 49% (IQR, 23.3%–65.7%) and had a median change in muscle mass of –3% (95% CI, –18.7) by day 7. Finally, a third study defined energy intake as the percentage intake of recommended dietary allowances; they found a mean energy intake of 73% (95% CI, 62%–84%) and a mean muscle mass loss of –13.33 ± 13.56%.^11^

### Risk factors for worse muscle mass loss

The main risk factors that were identified in the studies were: length of PICU stay, length of mechanical ventilation, length of hospital stay, corticosteroid use, neuromuscular blockade use, and maximal C‐reactive protein (CRP) value. Length of PICU stay was reported in seven out of eight studies,^7^, ^8^, ^9^, ^10^, ^11^, ^12^, ^13^ with a median value of 10 days (range, 3.9–14). The study involving patients with the longest PICU stay (median of 14 days [IQR, 11–25]) found a muscle mass change of –13% (IQR, –24 to –0.5%). However, the study with a median stay of 3.9 days (IQR, 2.0–12.3 days) had a mean muscle mass of −6.2% (95% CI, −21.9 to 9.4%).

All studies reported length of mechanical ventilation, with an overall median value of 7.2 days (range, 1.4–12).^7^, ^8^, ^9^, ^10^, ^11^, ^12^, ^13^, ^14^ The study involving children with the longest length of ventilation (median of 12 days IQR, 7–23), had a mean muscle mass loss of –8.2 ± 8.0%. The study with the median mechanical ventilation length of 1.4 days (IQR, 0.1–8.0) found a mean muscle decrease of –6.2% (95% CI, –21.8 to 9.8%).

Length of hospital stay was reported in four studies with a median value of 25.6 days (range, 15.7–30).^8^, ^10^, ^13^, ^14^ The muscle mass change in the study that had a median length of 30 days (IQR, 18–50) was a median of –13% (IQR, –24 to –0.5%), whereas the study with a length of stay of 15.7 (IQR, 8.87–25.75) days had a median muscle mass change of –3% (IQR, –18.7%).

Three out of eight studies reported neuromuscular blockade (NMB) use, in which 47 (44.6%) of children received neuromuscular blockade.^8^, ^10^, ^13^ Three studies with 17 + 13 participants who received NMB, reported the mean muscle mass change was −8.62 (95% CI, −15.7 to −1.54%), or a median change of −3 (IQR, −18.7%) to −13% (IQR −24 to −0.5%).^7^, ^9^, ^12^

Maximal CRP was reported in three studies.^7^, ^8^, ^13^ The overall median value of maximal CRP value was 86 mg/L (range, 41.8–139 mg/L), in three out of eight of the publications.^8^, ^10^, ^13^ The study with the median maximal CRP of 139 mg/L (IQR, 61–203 mg/L) had a median change in muscle mass of −13% (IQR, −24 to −0.5%), whilst the study with the lowest median CRP value of 41.8 mg/L (IQR, 19.5–136.9 mg/L) found a lower median change in muscle mass of −3% (IQR, −18.7%).

### Quality assessment

**Figure 4:**
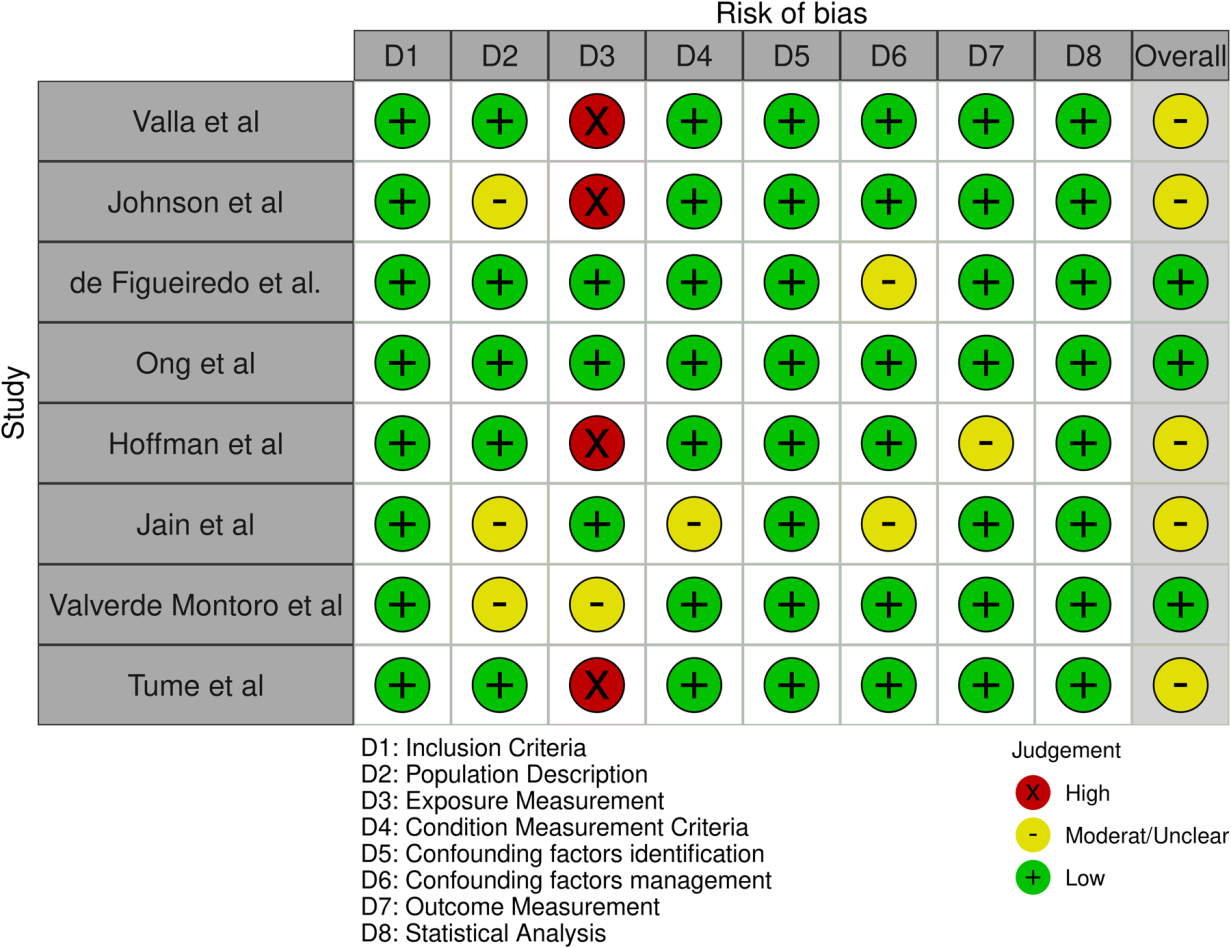
Risk of bias on individual studies assessed by Joanna Briggs Institute Critical Appraisal Checklist for Analytical cross‐sectional studies^20^: author's judgments for each included study.

All observational studies were considered to be of low‐moderate risk of bias based on the JBI tool.^20^ The results of the quality assessment are presented in Figure 4.

## DISCUSSION

This is the first systematic review to summarize muscle mass changes in children who are critically ill. These results show that the median muscle mass loss from admission to days 5–7 in children who are critically ill was 9.8%. These results also show that almost half (49.2%) of participants developed muscular atrophy (defined as >10%) during their PICU stay. These results are consistent with those in adults who are critically ill, in that they found adults lost on average approximately 2% of skeletal muscle per day during their first week of intensive care stay.^25^

Nutrition is thought to play a crucial role in maintaining muscle mass^26^ because of its effect on muscle protein synthesis and muscle protein turnover found in animal studies. A systematic review by Ong et al.^26^ also found that, once nutrition rehabilitation was commenced, the decreased muscle protein synthesis was reversed. In the current review, all studies that reported protein intake observed lower intakes in comparison with the recommended 1.5 g/kg/day and a decrease in muscle mass.^27^, ^28^ Similar results were observed for energy intake. These findings highlight the challenge of providing adequate protein and energy intake in this population. Despite existing nutrition guidelines,^29^ adequate intakes are still difficult to achieve for several reasons, such as enteral intolerance, electrolyte abnormalities, fasting for procedures. Strategies such as the presence of a nutrition team in PICU, education, or the use of nutrition protocol, are necessary.

It should be noted that, although sufficient protein intake is required for the maintenance of muscle mass, in the absence of mechanical stimulation from activity, protein and energy intake alone have not been shown to prevent atrophy in bedrest studies.^30^, ^31^ PICS‐p can result in physical changes, including muscular weakness, which can impair children's recovery and rehabilitation.^2^, ^3^ This study has described that muscular mass loss does occur in a large proportion of the pediatric critical care population. This is an issue increasingly being recognized in pediatric critical care, and early mobilization or early rehabilitation interventions are being instituted in many PICUs internationally. A systematic review found that, despite the definition of early mobilization varying, it is safe and feasible in this population.^32^ A more recent study showed more promising results and suggested starting mobilization within the first 72 h of PICU admission to have the best outcome.^33^

All studies in this review used a substitute of either quadriceps femoris muscular thickness or the cross‐sectional area of one of these muscles to measure the muscle mass. Valla et al.^7^ validated this measurement technique of quadriceps femoris muscular thickness in children. Only three of these eight studies used this validated technique. Seven out of eight studies measured the quadricep thickness, whereas one study measured the cross‐sectional area of the rectus femoris muscle. All studies excluded participants who had neuromuscular disease at admission, improving the consistency of results.

All studies described the exact measurement location; however, this was inconsistent, which could introduce bias and produce different muscle thickness values. However, because the location was consistent within each study, the magnitude of change reported in each study should not be notably affected. Furthermore, on protein intake and energy intake, only four and three of the studies, respectively, reported these values, and each of the studies had a different definition for intake. Thus, there are likely to be other variables that impact muscle mass/change that were not evaluated. Nutrition was evaluated crudely in the studies and not in the context of individual patient intakes compared with requirements based on their specific age and medical conditions. No studies have determined protein requirements using the nitrogen balance method, which is considered the gold standard^34^ Although protein intake is important, sufficient energy is also required to utilize the protein for muscle synthesis, and this review is limited by the inconsistent and crude reporting of nutrition intake in the studies and the determinations of both protein and energy requirements.

Overall, the review is limited by the quality of the evidence available. All the studies are observational studies, and subjective to confounding and bias. Another limitation of this review was the inability to conduct a meta‐analysis on the impact of each risk factor on muscle mass because of the differing definitions used. A core outcome set for PICU studies involving nutrition interventions is urgently required to improve the reporting and consistency in future studies. Finally, only two out of eight studies followed up with patients after PICU discharge. In the two studies that did follow‐up, the time varied from 3 to 6 months, and attrition was high, reducing numbers even further.^8^, ^12^ Therefore, the long‐term impact of muscle mass in critical care remains unknown. Further studies do need to consistently follow‐up children for at least 6 months after critical illness, using age‐validated functional assessment scores as well as muscle ultrasound. In summary, the methods of measurement of the quadriceps muscle and the studies’ results reporting limited the data we could analyze. Despite these limitations, this is the first systematic review to collate and analyze the published studies, clearly identifying the gaps and limitations in the current research and suggesting what future research needs to address.

## CONCLUSIONS

This is the first systematic review to examine muscle mass changes in children who are critically ill and showed around a 10% decrease in muscle mass, with half of children experiencing a decrease in muscle mass loss of >10% during their PICU stay. However, these results are based on cohort studies, which are subject to confounding. Evidence from adults who are critically ill does not suggest a benefit of higher calories and/or protein doses in improving outcomes. More adequately powered randomized studies with longer‐term follow‐up (using consistent definitions) are required to understand this issue further in children.

## AUTHOR CONTRIBUTIONS

Lyvonne N. Tume and Frederic V. Valla conceived the study; Lyvonne N. Tume, Frederic V. Valla, Paul Comfort, Corinne Jotterand Chaparro, and Lynne Latten designed the study; Lewis J. Stacey led the acquisition of data; Lewis J. Stacey undertook data collection; Lewis J. Stacey, Chao Huang, and Lyvonne N. Tume analyzed the data; Lyvonne N. Tume, Frederic V. Valla, Corinne Jotterand Chaparro, Paul Comfort, Chao Huang, and Lynne Latten contributed equally to the interpretation of the data; Lewis J. Stacey drafted the manuscript; and all authors critically revised the manuscript, agree to be fully accountable for ensuring the integrity and accuracy of the work, and read and approved the final manuscript.

## CONFLICT OF INTEREST STATEMENT

The authors declare no conflicts of interest.

## Supporting information

Supporting information Table S1.

Supporting information Table S2.

Supporting information Table S3.
