# Supplementary material for: The relationship between muscle mass changes and protein or energy intake in critically ill children: A systematic review and meta‐analysis
Source: JPEN J Parenter Enteral Nutr. 2024 Dec 24;49(2):152–64. doi: 10.1002/jpen.2715 (PMC11794675; doi:10.1002/jpen.2715)
Supplement: Supplementary file 1 — Supporting information Table S1. [file JPEN-49-152-s002.pdf]

Wed, July 31, 2024 11:07:32 am

#	Query	Limiters/Expanders	Last Run Via	Results
S15	S3 AND S6 AND S9	Limiters - Publication Date: 20100101-20241231<br>Expanders - Apply equivalent subjects<br>Narrow by SubjectAge: - all child: 0-18 years<br>Search modes - Find all my search terms	Interface - EBSCOhost Research Databases<br>Search Screen - Advanced Search<br>Database - MEDLINE	22
S14	S3 AND S6 AND S9	Limiters - Publication Date: 20100101-20241231<br>Expanders - Apply equivalent subjects<br>Search modes - Find all my search terms	Interface - EBSCOhost Research Databases<br>Search Screen - Advanced Search<br>Database - MEDLINE	246
S13	S3 AND S6 AND S9 AND S12	Limiters - Publication Date: 20100101-20241231<br>Expanders - Apply equivalent subjects<br>Search modes - Find all my search terms	Interface - EBSCOhost Research Databases<br>Search Screen - Advanced Search<br>Database - MEDLINE	21
S12	S10 OR S11	Expanders - Apply equivalent subjects<br>Search modes - Find all my search terms	Interface - EBSCOhost Research Databases<br>Search Screen - Advanced Search<br>Database - MEDLINE	2,670,719
S11	(MM "Infant") OR (MM "Infant, Newborn") OR OR (MM "Adolescent")	Expanders - Apply equivalent subjects<br>Search modes - Find all my search	Interface - EBSCOhost Research Databases<br>Search Screen - Advanced Search	2,320

	OR (MM "Child, Preschool") or (MM "Child") OR (OR (MM "Child, Preschool")	terms	Database - MEDLINE	
S10	AB (Child* or Newborn* or Infant* or Toddler* or Boy* or Girl* or Neonate* or Teen* or Adolescent* or P#ediatric*) OR TI (Child* or Newborn* or Infant* or Toddler* or Boy* or Girl* or Neonate* or Teen* or Adolescent* or P#ediatric*)	Expanders - Apply equivalent subjects Search modes - Find all my search terms	Interface - EBSCOhost Research Databases Search Screen - Advanced Search Database - MEDLINE	2,670,164
S9	S7 OR S8	Expanders - Apply equivalent subjects Search modes - Find all my search terms	Interface - EBSCOhost Research Databases Search Screen - Advanced Search Database - MEDLINE	74,410
S8	(MM "Sarcopenia") OR (MM "Muscle Hypotonia") OR (MM "Muscle Hypertonia") OR (MM "Muscular Atrophy") OR (MM "Atrophy")	Expanders - Apply equivalent subjects Search modes - Find all my search terms	Interface - EBSCOhost Research Databases Search Screen - Advanced Search Database - MEDLINE	23,897
S7	AB (“Musc* mass” or “musc* atrophy” or “Musc* wast*” or “Musc* thickness” or "Musc* size" or Sarcopenia* or “Quadricep* size” or “Quadricep* thickness” or “Quadricep* wasting” or “Quadricep* atrophy” or “Quadricep* wast*”) OR TI (“Musc* mass” or “musc* atrophy” or “Musc* wast*” or “Musc* thickness” or "Musc* size" or Sarcopenia* or “Quadricep* size” or “Quadricep* thickness” or “Quadricep*	Expanders - Apply equivalent subjects Search modes - Find all my search terms	Interface - EBSCOhost Research Databases Search Screen - Advanced Search Database - MEDLINE	64,612

	wasting" or "Quadricep* atrophy" or "Quadricep* wast*")			
S6	S4 OR S5	Expanders - Apply equivalent subjects<br>Search modes - Find all my search terms	Interface - EBSCOhost Research Databases<br>Search Screen - Advanced Search<br>Database - MEDLINE	454,227
S5	(MM "Ultrasonography") OR (MM "Quadriceps Muscle")	Expanders - Apply equivalent subjects<br>Search modes - Find all my search terms	Interface - EBSCOhost Research Databases<br>Search Screen - Advanced Search<br>Database - MEDLINE	55,612
S4	AB (("Quadricep* femoris") w3 (ultrasonography or ultrasound*) or (Quadricep*) w3 (ultrasonography or ultrasound*) or (thigh) w3 (ultrasonography or ultrasound*) or "Quadricep* femoris ultrasound*" or "Quadricep* femoris ultrasonography" or "Thigh ultrasound*" or "Thigh ultrasonography" or ultrasound* or ultrasonography or sonography) OR TI (("Quadricep* femoris") w3 (ultrasonography or ultrasound*) or (Quadricep*) w3 (ultrasonography or ultrasound*) or (thigh) w3 (ultrasonography or ultrasound*) or "Quadricep* femoris ultrasound*" or "Quadricep* femoris ultrasonography" or "Thigh ultrasound*" or "Thigh ultrasonography" or ultrasound* or ultrasonography or sonography)	Expanders - Apply equivalent subjects<br>Search modes - Find all my search terms	Interface - EBSCOhost Research Databases<br>Search Screen - Advanced Search<br>Database - MEDLINE	435,647
S3	S1 OR S2	Expanders - Apply equivalent subjects<br>Search modes - Find all my search	Interface - EBSCOhost Research Databases<br>Search Screen - Advanced Search	330,138

		terms	Database - MEDLINE	
S2	(MM "Intensive Care, Neonatal") OR (MM "Hospitalization") OR (MM "Critical Care") OR (MM "Critical Illness") OR (MM "Intensive Care Units, Neonatal") (MM "Intensive Care Units, Pediatric")	Expanders - Apply equivalent subjects<br>Search modes - Find all my search terms	Interface - EBSCOhost Research Databases<br>Search Screen - Advanced Search<br>Database - MEDLINE	104,406
S1	AB (“Critical care” or “P#ediatric Critical Care” or “Intensive care” or “P#ediatric Intensive care” or PICU or ICU or “Critically ill child”) OR TI (“Critical care” or “P#ediatric Critical Care” or “Intensive care” or “P#ediatric Intensive care” or PICU or ICU or “Critically ill child”)	Expanders - Apply equivalent subjects<br>Search modes - Find all my search terms	Interface - EBSCOhost Research Databases<br>Search Screen - Advanced Search<br>Database - MEDLINE	265,522

EBSCOhost

Wed, July 31, 2024 11:15:59 am

#	Query	Limiters/Expanders	Last Run Via	Results
S15	S3 AND S6 AND S9	Limiters - Publication Date: 20100101-20241231<br>Expanders - Apply equivalent subjects<br>Narrow by SubjectAge: - all child<br>Search modes - Find all my search terms	Interface - EBSCOhost Research Databases<br>Search Screen - Basic Search<br>Database - CINAHL Complete	11
S14	S3 AND S6 AND S9	Limiters - Publication Date: 20100101-20241231<br>Expanders - Apply equivalent subjects<br>Search modes - Find all my search terms	Interface - EBSCOhost Research Databases<br>Search Screen - Basic Search<br>Database - CINAHL Complete	106
S13	S3 AND S6 AND S9 AND S12	Limiters - Publication Date: 20100101-20241231<br>Expanders - Apply equivalent subjects<br>Search modes - Find all my search terms	Interface - EBSCOhost Research Databases<br>Search Screen - Basic Search<br>Database - CINAHL Complete	11
S12	S10 OR S11	Expanders - Apply equivalent subjects<br>Search modes - Find all my search terms	Interface - EBSCOhost Research Databases<br>Search Screen - Basic Search<br>Database - CINAHL Complete	932,982
S11	(MM "Infant") OR (MM "Infant, Newborn") OR OR (MM "Adolescent") OR (MM "Child, Preschool") or (MM	Expanders - Apply equivalent subjects<br>Search modes - Find all my search terms	Interface - EBSCOhost Research Databases<br>Search Screen - Basic Search<br>Database - CINAHL Complete	1,188

	"Child") OR (OR (MM "Child, Preschool")			
S10	AB (Child* or Newborn* or Infant* or Toddler* or Boy* or Girl* or Neonate* or Teen* or Adolescent* or P#ediatric*) OR TI (Child* or Newborn* or Infant* or Toddler* or Boy* or Girl* or Neonate* or Teen* or Adolescent* or P#ediatric*)	Expanders - Apply equivalent subjects<br>Search modes - Find all my search terms	Interface - EBSCOhost Research Databases<br>Search Screen - Basic Search<br>Database - CINAHL Complete	932,519
S9	S7 OR S8	Expanders - Apply equivalent subjects<br>Search modes - Find all my search terms	Interface - EBSCOhost Research Databases<br>Search Screen - Basic Search<br>Database - CINAHL Complete	19,616
S8	(MM "Sarcopenia") OR (MM "Muscle Hypotonia") OR (MM "Muscle Hypertonia") OR (MM "Muscular Atrophy") OR (MM "Atrophy")	Expanders - Apply equivalent subjects<br>Search modes - Find all my search terms	Interface - EBSCOhost Research Databases<br>Search Screen - Basic Search<br>Database - CINAHL Complete	7,544
S7	AB (“Musc* mass” or “musc* atrophy” or “Musc* wast*” or “Musc* thickness” or "Musc* size" or Sarcopenia* or “Quadricep* size” or “Quadricep* thickness” or “Quadricep* wasting” or “Quadricep* atrophy” or “Quadricep* wast*”) OR TI (“Musc* mass” or “musc* atrophy” or “Musc* wast*” or “Musc* thickness” or "Musc* size" or Sarcopenia* or “Quadricep* size” or “Quadricep* thickness” or “Quadricep* wasting” or “Quadricep* atrophy” or “Quadricep* wast*”)	Expanders - Apply equivalent subjects<br>Search modes - Find all my search terms	Interface - EBSCOhost Research Databases<br>Search Screen - Basic Search<br>Database - CINAHL Complete	17,154

S6	S4 OR S5	Expanders - Apply equivalent subjects<br>Search modes - Find all my search terms	Interface - EBSCOhost Research Databases<br>Search Screen - Basic Search<br>Database - CINAHL Complete	104,802
S5	(MM "Ultrasonography") OR (MM "Quadriceps Muscle")	Expanders - Apply equivalent subjects<br>Search modes - Find all my search terms	Interface - EBSCOhost Research Databases<br>Search Screen - Basic Search<br>Database - CINAHL Complete	22,022
S4	AB ((“Quadricep* femoris”) w3 (ultrasonography or ultrasound*) or (Quadricep*) w3 (ultrasonography or ultrasound*) or (thigh) w3 (ultrasonography or ultrasound*) or “Quadricep* femoris ultrasound*” or "Quadricep* femoris ultrasonography" or “Thigh ultrasound*” or "Thigh ultrasonography" or ultrasound* or ultrasonography or sonography) OR TI ((“Quadricep* femoris”) w3 (ultrasonography or ultrasound*) or (Quadricep*) w3 (ultrasonography or ultrasound*) or (thigh) w3 (ultrasonography or ultrasound*) or “Quadricep* femoris ultrasound*” or "Quadricep* femoris ultrasonography" or “Thigh ultrasound*” or "Thigh ultrasonography" or ultrasound* or ultrasonography or sonography)	Expanders - Apply equivalent subjects<br>Search modes - Find all my search terms	Interface - EBSCOhost Research Databases<br>Search Screen - Basic Search<br>Database - CINAHL Complete	99,635
S3	S1 OR S2	Expanders - Apply equivalent subjects<br>Search modes - Find all my search terms	Interface - EBSCOhost Research Databases<br>Search Screen - Basic Search<br>Database - CINAHL Complete	149,981

S2	(MM "Intensive Care, Neonatal") OR (MM "Hospitalization") OR (MM "Critical Care") OR (MM "Critical Illness") OR (MM "Intensive Care Units, Neonatal") (MM "Intensive Care Units, Pediatric")	Expanders - Apply equivalent subjects<br>Search modes - Find all my search terms	Interface - EBSCOhost Research Databases<br>Search Screen - Basic Search<br>Database - CINAHL Complete	49,734
S1	AB (“Critical care” or “P#ediatric Critical Care” or “Intensive care” or “P#ediatric Intensive care” or PICU or ICU or “Critically ill child”) OR TI (“Critical care” or “P#ediatric Critical Care” or “Intensive care” or “P#ediatric Intensive care” or PICU or ICU or “Critically ill child”)	Expanders - Apply equivalent subjects<br>Search modes - Find all my search terms	Interface - EBSCOhost Research Databases<br>Search Screen - Basic Search<br>Database - CINAHL Complete	118,429

Embase <1974 to 2024 July 30>

1 ("Critical care" or "P?ediatric Critical Care" or "Intensive care" or "P?ediatric Intensive care" or PICU or ICU or "Critically ill child").mp. [mp=title, abstract, heading word, drug trade name, original title, device manufacturer, drug manufacturer, device trade name, keyword heading word, floating subheading word, candidate term word] 591924
2 *pediatric intensive care unit/ or *intensive care unit/ or *intensive care/ or *critical illness/ or *intensive care nursing/ 131116
3 1 or 2 596672
4 (("Quadricep* femoris" adj3 (ultrasonography or ultrasound*)) or (Quadricep* adj3 (ultrasonography or ultrasound*)) or (thigh adj3 (ultrasonography or ultrasound*)) or "Quadricep* femoris ultrasound*" or "Quadricep* femoris ultrasonography" or "Thigh ultrasound*" or "Thigh ultrasonography" or ultrasound* or ultrasonography or sonography).mp. [mp=title, abstract, heading word, drug trade name, original title, device manufacturer, drug manufacturer, device trade name, keyword heading word, floating subheading word, candidate term word] 834542
5 *quadriceps femoris muscle/ or *ultrasound/ or *skeletal muscle/ 123769
6 4 or 5 888439
7 ("Musc* mass" or "musc* atrophy" or "Musc* wast*" or "Musc* thickness" or Sarcopenia* or "Quadricep* size*" or "Quadricep* thickness" or "Quadricep* wasting" or "Quadricep* atrophy" or "Quadricep* wast*").mp. [mp=title, abstract, heading word, drug trade name, original title, device manufacturer, drug manufacturer, device trade name, keyword heading word, floating subheading word, candidate term word] 133543
8 *muscle atrophy/ or *muscle thickness/ or *muscle mass/ or *sarcopenia/ 31525
9 7 or 8 133543
10 (Child* or Newborn* or Infant* or Toddler* or Boy* or Girl* or Neonate* or Teen* or Adolescent* or P?ediatric*).mp. [mp=title, abstract, heading word, drug trade name, original title, device manufacturer, drug manufacturer, device trade name, keyword heading word, floating subheading word, candidate term word] 5155082
11 *child/ or *infant/ or *neonate/ or *preschool child/ or *pediatrics/ or *baby/ or *adolescent/ 195881
12 10 or 11 5155418
13 3 and 6 and 9 and 12 59

Search Name: PICU US Critical Care Study
Date Run: 31/07/2024 12:19:37
Comment:

ID Search Hits
#1 (“Critical care” or P?ediatric NEXT Critical NEXT Care or “Intensive care” or P?ediatric NEXT Intensive NEXT care or PICU or ICU or “Critically ill child”):ti,ab,kw OR (“Critical care” or P?ediatric NEXT Critical NEXT Care or “Intensive care” or P?ediatric NEXT Intensive NEXT care or PICU or ICU or “Critically ill child”):ti,ab,kw (Word variations have been searched) 51096
#2 MeSH descriptor: [Critical Care] explode all trees 3101
#3 MeSH descriptor: [Critical Illness] explode all trees 3712
#4 MeSH descriptor: [Intensive Care Units, Pediatric] explode all trees 1751
#5 #1 or #2 or #3 or #4 52025
#6 (Quadricep* NEXT femoris) w3 (ultrasonography or ultrasound*) or (thigh) w3 (ultrasonography or ultrasound*) or Quadricep* NEXT femoris NEXT ultrasound* or Quadricep* NEXT femoris NEXT ultrasonography or Thigh NEXT ultrasound* or "Thigh ultrasonography" or ultrasound* or ultrasonography or sonography 61526
#7 MeSH descriptor: [Ultrasonography] explode all trees 19452
#8 MeSH descriptor: [Quadriceps Muscle] explode all trees 1284
#9 #6 or #7 or #8 68202
#10 Musc* NEXT mass or Musc* NEXT atrophy or Musc* NEXT wast* or Musc* NEXT thickness or Musc* NEXT size or Sarcopenia* or Quadricep* NEXT size* or Quadricep* NEXT thickness or Quadricep* NEXT wasting or Quadricep* NEXT atrophy or Quadricep* NEXT wast* 10636
#11 MeSH descriptor: [Muscular Atrophy] explode all trees 1437
#12 MeSH descriptor: [Sarcopenia] explode all trees 936
#13 #10 or #11 or #12 10636
#14 Child* or Newborn* or Infant* or Toddler* or Boy* or Girl* or Neonate* or Teen* or Adolescent* or P?ediatric* 393030
#15 MeSH descriptor: [Child] explode all trees 82500
#16 MeSH descriptor: [Infant] explode all trees 46407

Electronic Supplementary File 1*: Search strings for our search for the following databases: MEDLINE (EMBSCOhost), CINAHL Complete (EMBSCOhost), EMBASE (Ovid) and CENTRAL (Cochrane), respectivel*
