# Supplementary material for: The relationship between muscle mass changes and protein or energy intake in critically ill children: A systematic review and meta‐analysis
Source: JPEN J Parenter Enteral Nutr. 2024 Dec 24;49(2):152–64. doi: 10.1002/jpen.2715 (PMC11794675; doi:10.1002/jpen.2715)
Supplement: Supplementary file 2 — Supporting information Table S2. [file JPEN-49-152-s001.pdf]

Study	Change in muscle mass % day 5-7, Median (IQR)	Change in muscle mass % day 5-7, mean (95% CI) or mean ±SD	Muscular atrophy, >10% muscle mass loss, N (%)	Protein intake, at day 5-7, median (IQR)	Protein intake at day 5-7, mean (95% CI)
Valla et al, 2017	-9.8 (-13.7 - -0.5), **day 5**		6 (35%)	-58.9% (-74.4/-28.0), **day 5, deficit**	
de Figeurdo et al, 2021		-12.85 ±14.07, **day 7**<br>-13.81 ±13.05, **day 7**	32 (58.2%)		**day 5** 59% (43/70) OR **day 6** 61% (50-74) OR **day 7** 72% (67-83)
Valverde Montoro et al, 2023	-13 (-24 - -0.5), **day 7**		23 (56%)		-0.34 (-1/0) g/kg/day, **day 7, deficit**
Tume et al, 2024	-3 (95%CI, -18.7), **day 7**		15 (44%)	45% (13.2-71), **day 5** OR 55% (20.2/68.2), **day 7**	

Electronic Supplementary File 2: *A table showing the percentage muscle mass change as either median (IQR) or mean (95% CI) or mean* ±SD and muscular atrophy N (%) with protein intake as a percentage or true value either as mean (IQR) or mean (95% CI).
