# Supplementary material for: The relationship between muscle mass changes and protein or energy intake in critically ill children: A systematic review and meta‐analysis
Source: JPEN J Parenter Enteral Nutr. 2024 Dec 24;49(2):152–64. doi: 10.1002/jpen.2715 (PMC11794675; doi:10.1002/jpen.2715)
Supplement: Supplementary file 3 — Supporting information Table S3. [file JPEN-49-152-s003.pdf]

Study	Change in muscle mass % day 5-7, Median (IQR)	Change in muscle mass % day 5-7, mean (95% CI) or mean ±SD	Muscular atrophy, >10% muscle mass loss, N (%)	Energy intake, % at day 5-7, median (IQR)	Energy intake % at day 5-7, mean (95% CI)
Valla et al, 2017	-9.8 (-13.7 - -0.5), **day 5**		6 (35%)	-55.3% (-64.0/-16.5), **day 5 deficit**	
de Figeurdo et al, 2021		-12.85 ±14.07, **day 7**<br>-13.81 ±13.05, **day 7**	32 (58.2%)		**day 5** 73% (62/84) OR **day 6** 80% (72/92) OR **day 7** 82% (73/95)
Tume et al, 2024	-3 (95% CI, -18.7), **day 7**		15 (44%)	36.5% (15.7-57), **day 5** OR 49% (23.2/65.7), **day 7**	

Electronic Supplementary File 3: *A table showing the percentage muscle mass change as either median (IQR) or mean (95% CI) or mean* ±SD and muscular atrophy N (%) with energy intake as a percentage either as mean (IQR) or mean (95% CI).
